# Business Strategy as Human Rights Risk: the Case of Private Equity

**DOI:** 10.1007/s12142-023-00680-w

**Published:** 2023-03-23

**Authors:** David Birchall, Nadia Bernaz

**Affiliations:** 1 London South Bank University, London, UK; 2 Wageningen University Law Group, Wageningen, Netherlands

**Keywords:** Private equity, Business and human rights, UN Guiding Principles on Business and Human Rights, Law and political economy, Human rights

## Abstract

In this article, we apply the UN Guiding Principles on Business and Human Rights to the private equity (PE) business model. PE firms often adopt a controversial, ‘value extractive’, business model based on high debt and extreme cost-cutting to generate investor returns. PE firms own large numbers of companies, including in many rights-related sectors. The model is linked to increased human rights risks to workers, housing tenants, and in privatized health and social care. We map these risks and analyse the human rights responsibilities of PE firms. Our analysis has major implications for understandings of human rights responsibility. We argue that value extractive methods are the root cause of eventual harm to human rights, even though they may not harm rights directly. To respect human rights, PE firms must mitigate the risks of these value extractive methods. We define how human rights due diligence (HRDD) could achieve this and argue that given the extent of harm and the lack of a business case for adopting such a view of human rights responsibility, business strategy level HRDD should be a core component of forthcoming HRDD laws.

## Introduction

Private equity (PE) firms adopt a business model based on attracting investors in order to purchase companies, which they then manage with the singular aim of delivering short-term value for those investors. As of June 2021, PE firms held $3 trillion in assets (Wigglesworth 2021). One in every 14 US workers works for PE-owned companies (Levintova 2020). Aggregating the portfolio companies of the five largest PE firms, these firms would be the largest employer in Europe and the second largest employer in the USA (Boston Consulting Group 2017). PE activity is increasing, particularly in many socio-economic rights–related business sectors, including healthcare and housing (Appelbaum and Batt 2020).

PE firms share several otherwise unusual and controversial business strategies. These include leveraged buyouts (LBOs), dividend recapitalizations, cost-cutting, and sale-and-leaseback. An LBO is a takeover partially funded by debt taken against the purchased entity. A dividend recapitalization is a further loan taken solely to pay investor dividends. Assets such as property will often be sold and leased back to fund further dividends. These practices are termed ‘value extraction’ (Froud and Williams 2007) when they deliver investor returns by weakening the company. Such practices can cause severe risks to workers and to dependent service users in housing, healthcare, prisons, and nursing homes, explored below.

Research has focused on the economics of PE (Ayash and Rastadz 2021) and some on the ethics of, and harm caused by, PE (Watt 2008; Morrell and Clark 2010; Appelbaum and Batt 2014). No research has been conducted into their compliance, or otherwise, with business and human rights (BHR) standards. This is a significant gap, because PE is widely criticized for the harm it causes to human rights (Appelbaum and Batt 2014). As such, they may cause adverse human rights impacts as defined under the UN Guiding Principles on Business and Human Rights (UNGPs).

This paper is the first to apply BHR principles to private equity business strategy. This is significant because of the size, distinctiveness, and alleged harmfulness of PE. The major conceptual move herein is to go beyond showing that specific harmful outcomes of PE investments breach human rights standards, but to show how the underlying model makes these harms inevitable, and therefore to build a case that the PE model itself should be considered a human rights risk. In analysing the application of BHR principles to the PE model, the paper also questions the scope and limits of human rights principles in managing, or at least reducing, harm, within the contemporary economy. This focus on business strategy develops nascent work on how underlying business strategy and incentives can increase risks to rights-holders (OHCHR 2021, 13; Shift 2021; Birchall 2021a, b; Birchall 2022a, b, 406–408), by focusing on a model in which business success is determined by a particularly narrow focus on investor returns.

We make this contribution through a comprehensive analysis of the value extractive PE business model, and the application of BHR principles to this model. Through methodical plotting of causal links from the business strategy to the harmful outcomes that breach the UNGPs, we demonstrate the innate risks to rights within the model. We demonstrate also that the key intra-corporate human rights risk management tool proposed by the UNGPs, that of human rights due diligence (HRDD), is conceptually capable of addressing these risks, but must be enacted prior to value extractive activities such as a dividend recapitalization because these activities cause foreseeable risk of harm to rights-holders. Because this rights protection is likely to significantly harm the profitability of PE activity, this use of HRDD should be mandated within forthcoming HRDD laws, such as that proposed by the EU (European Commission 2022). Through this argument, we provide an original interpretation of the UNGPs designed to address structural economic concerns rather than only the end-result of human rights abuses. This could engender a significant shift towards a more comprehensive form of BHR capable of addressing the root causes of human rights harm.

This article proceeds by first introducing our normative framework, whose main element is the UNGPs’ second pillar, the corporate responsibility to respect human rights, and elaborating the meaning of some key concepts and tools under that pillar. It then presents the PE strategy by identifying the core elements of this business model. ‘The Human Rights Impacts of the PE Model’ evidences harmful outcomes within PE caused by the PE business strategy, and contextualizes them as breaches of PE firms’ human rights responsibility. ‘Private Equity: Defining the Problem in Human Rights Terms’ defines how the UNGPs can be applied to the root business strategy of PE firms. The final section concludes with three principles designed to engender a meaningful shift within BHR thought towards root causes such as business strategy.

## Normative Framework

In ‘The Human Rights Impacts of the PE Model’ and ‘Private Equity: Defining the Problem in Human Rights Terms’ of this article, we evaluate PE firms’ corporate behaviour and human rights impacts against our normative framework. The main element of this framework is the UNGPs’ second pillar: the corporate responsibility to respect human rights. The UNGPs are most authoritative business and human rights (BHR) standard. They were endorsed by the United Nations (UN) Human Rights Council in 2011. They implement the ‘Protect, Respect, Remedy’ framework designed by John Ruggie in 2008 (United Nations 2008), under which states have a duty to protect human rights, corporations have a responsibility to respect human rights, and both groups have differentiated responsibilities to provide access to remedy in case of breach.

The corporate responsibility to respect is limited to a responsibility to ‘do no harm’ rather than to realize rights, but this is understood broadly (Mares 2014). Companies hold responsibilities to proactively avoid causing harm, including through risk assessments such as human rights due diligence (HRDD). ‘Respect’ is defined according to Principle 13 of the UNGPs as a responsibility to avoid causing, contributing to, or being linked to through business relationships any ‘adverse human rights impacts’. ‘Adverse human rights impacts’ are defined in the Office of the High Commissioner for Human Rights (OHCHR) guidance on the corporate responsibility to respect as occurring ‘when an action removes or reduces the ability of an individual to enjoy his or her human rights’ (OHCHR 2021, 5; Birchall 2019a, b, c).

This definition incorporates both elements of the state obligation to respect human rights in international law, consisting of a prohibition on state interference with existing access or enjoyment of human rights, and second, a more positive state duty ‘to ensure that existing access is not disrupted’ (Nolan and Dutschke 2010, 282–3). ‘Interference’ broadly covers proactive violations of rights, such as a company employing child labour. Ensuring that existing access is not disrupted is relevant to companies that supply or manage essential resources. A company engaged in healthcare provision has a responsibility to ensure its actions do not disrupt access to healthcare for dependent individuals on the grounds that such a disruption would ‘remove or reduce’ those individuals’ enjoyment of rights (Macchi et al. forthcoming).

The corporate responsibility to respect incorporates ‘internationally recognized human rights’, as well as ‘additional standards’ in relevant contexts, including minority protections and international humanitarian law (UNGPs, Principle 12). Internationally recognized human rights refers to, “at a minimum” the rights protected under the Universal Declaration of Human Rights, the 1966 International Covenant on Civil and Political Rights, and Economic, Social and Cultural Rights (ICESCR), and the International Labour Organization’s Declaration on Fundamental Principles and Rights at Work. In the past decades, the two Covenants’ dedicated treaty bodies, the Human Rights Committee and the Committee on Economic Social and Cultural Rights, have issued ‘authoritative interpretations’ (Mechlem 2009, 929–930) of some of the Covenants’ provisions, mainly through the publications of General Comments. These interpretations are technically non-binding, since state parties have not agreed to be bound by any particular contextual interpretation of treaties, but they represent the standard interpretation with which states should comply absent any strong counterargument. Interpretation is necessary because the Covenants themselves provide no details on the substantive content of human rights, for example, the scope and limits of ‘the right to health’. General Comments address state obligations but businesses should respect rights along the same substantive metrics. Both states and businesses should respect the right to health, and this includes metrics of non-discrimination, adequacy and affordability (CESCR, General Comment 14, para. 12). States also hold wider obligations to respect, protect, and fulfil human rights. States must ‘progressively realize’ universal access to healthcare within their jurisdiction, while businesses must ensure that they do not ‘deprive’ any individuals of access to adequate, affordable, non-discriminatory healthcare.

We include those authoritative interpretations in our normative framework. Specifically, in this paper, we interpret the content of rights under the UNGPs — their scope, limits, and inclusions — following General Comments of the relevant treaty body, primarily the Committee on Economic, Social and Cultural Rights (CESCR), the body that oversees the International Covenant on Economic, Social and Cultural Rights (ICESCR).

Finally, HRDD is the primary tool through which companies should address their human rights risks (UNGPs, Principles 17 & 18). HRDD is designed as an ongoing risk management process to ‘identify, prevent, mitigate and account’ for human rights risks (Bonnitcha and McCorquodale 2017). A human rights risk is defined as a potential human rights impact. Companies should analyse the possible risks within their operation and take action to eliminate or at least mitigate these risks. Where a company does cause a human rights impact, the affected individuals should have remediation provided through legitimate processes (Principle 22).

It is worth mentioning two forms of interplay between the state duty to protect and the corporate responsibility to respect. First, states hold an obligation to regulate corporations so as to ensure that they respect human rights. This entails eliminating opportunities for corporations to profit from harm, covering everything from implementing and enforcing a minimum wage, to ensuring that corporate law incentives work to promote rights-respectful business (UNGPs, Principle 3). Secondly, states hold obligations at the ‘state-business nexus’ to use work in rights-respectful ways alongside business, such as in public procurement, and corporations hold a responsibility to avoid complicity in state human rights violations (UNGPs Principles 6 and 17). This interplay becomes relevant in ‘Private Equity: Defining the Problem in Human Rights Terms’ of this paper, where the regulation of PE firms by states is addressed.

Research into the UNGPs covers numerous angles and industries (Wettstein 2015; Arnold 2016; Fasterling 2017). However, most applied research focuses on specific sectors such as factories, mining, and tourism (Baleva 2018; Seck 2011; Nolan 2017). Mining, for example, carries clear industry-specific risks, from pollution to worker safety. The UNGPs have also been applied to value chain business models (Bright et al. 2020), the on-demand economy (Natour 2016), and banking, itself based on a unique business model (De Felice 2015). Each focused on how the sector contains specific human rights risks, such as to labour rights in the on-demand economy. In 2021, consultancy company Shift published Business Model Red Flags, a set of indicators to help relevant stakeholders identify human rights risks associated with certain business models (Shift 2021). The red flags are divided into (1) red flags in the value proposition, i.e. what the company offers and to whom; (2) red flags in the value chain, i.e. how the company delivers value; and (3) red flags in the value model, i.e. how the business model is profitable. Our approach is similar but is focused on PE strategies of generating investor returns as a root cause of eventual harm to rights-holders. It is not that PE firms are particularly prone to investments in harmful products or using rights-abusive supply chain labour, rather it is that the value extractive model itself, wherever the investment is made, encourages rights-abusive patterns.

**Fig. 1:**
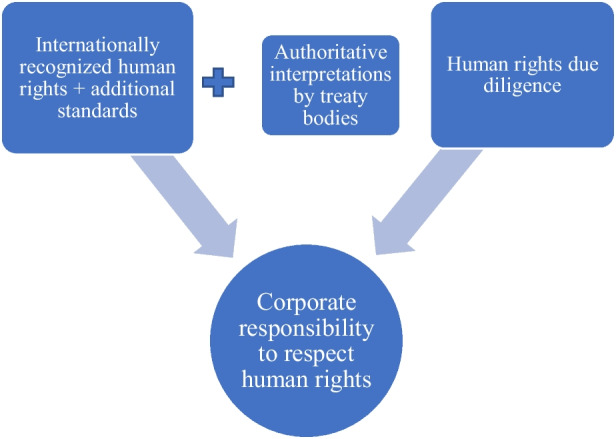
Normative framework

In sum, within pillar two, we differentiate between the procedural aspect, namely the human rights due diligence process companies are to undertake to avoid causing, contributing to or being linked to human rights abuse, and the substantive aspect, the rights businesses should respect, phrased as ‘internationally recognized human rights’ and additional standards. Those two aspects form the core of our normative framework. Additionally, we include treaty bodies’ authoritative interpretations in the framework, visualized in Fig. 1.

## Unpacking the PE Business Model

The business model of PE firms is as follows. PE firms specialize in buying and managing companies (Wright et al. 2009, 3). Particularly since quantitative easing following the financial crisis, the number of PE investments has exploded, including in human rights–related areas. PE firms are usually organized as limited partnerships. The managers of the PE fund act as General Partners, contributing a small amount of investment and managing the fund, and outside investors join as limited partners. The most common type of PE activity is to buy out companies, usually taking public companies into private ownership, particularly since the 1990s (Wright et al. 2013, 544), which the General Partners or their appointees usually then manage directly (Gong and Wu 2011, 195). This is a key distinction between PE and hedge funds or asset managers, who usually trade in shares, rarely involving themselves in company management. They promise rapid, high returns to investors and aim to divest of the company within 3–5 years (Morgan and Nasir 2021, 3).

It is argued that LBOs increase efficiency, using their management expertise to streamline operations (De Cock and Nyberg 2016). The strong financial returns to investors are often used to evidence this (Cumming et al. 2007, 441). Conversely, these efficiency savings often increase the risks of bankruptcy and adverse human rights impacts. It must be emphasized that LBOs are common and can work to a variety of ends. Froud and Williams (2007) distinguish between ‘value creation’ through LBOs, where General Partners rescue a struggling firm, improving its economic performance and profiting through that improvement, and ‘value extraction’, where investors take a profitable firm and strip it for quick gains. Wright et al. (2009, 3) summarize the alleged risks of the extractive form to include ‘asset stripping… profiting from the reselling of assets within short periods of time… instigating restructuring within firms that negatively impacts on employment and employee remuneration… [and] the use of leverage and offshore holding companies [that] reduces tax charges’. More recently, involvement in rights-related sectors such as healthcare has ballooned, causing additional specifically human rights–related risks (Elkind and Burke 2020). Different legal regimes affect the possibilities for LBOs significantly (Tuch 2020).

PE firms often target cash and asset-rich businesses for two reasons. First, to fund their initial LBO, with the purchase paid for, in part, with debt leveraged against the company being purchased, usually amounting to around two-thirds of the total price (Morgan and Nasir 2021). The LBO makes the purchase more affordable, but also reduces the tax to be paid by the once wealthy company, by turning taxable income into non-taxable debt servicing. Second, once the purchase is complete, assets can be sold to fund dividends, and further loans taken out against the company to fund more dividends. Naturally, the company that was worth $100 million and debt free now has around $70 million in debt to be serviced, potentially weakening its long-term stability. Often, because of the high (and subsequently increasing) level of debt, this debt will be low rated and attract higher interest rates (Batt and Morgan 2020, 88).

The PE business model can deliver profit for investors through improved company performance (Wright et al. 2009, 17). However, innate to the model is the severe risk of harm occurring, particularly as PE firms invest more in socio-economic rights–related areas. This is one reason why the human rights lens is useful. It promotes focus on human rights risks without prejudice to those investments that do not cause harm. The UNGPs do not obligate sweeping prohibitions, but that firms understand and manage their human rights risks. As such, the standards may be well-suited to a business model that contains both risks and opportunities.

Following LBOs, three methods of value extraction are most common: reduced operating costs, further debt loaded onto the company, and the sale or partitioning of valuable components. First, reduced operating costs are achieved through redundancies, and, where legally possible, less favourable working conditions and abrogation of collective bargaining agreements, denoted in one study as ‘a transfer from workers to PE investors’ (Appelbaum and Batt 2014, 73). Although the new managers cut most operating costs, they typically require portfolio companies to sign a Master Services Agreement in which the acquired company pays the PE firm various fees for services. One study of 592 LBO transactions found that PE firms extracted $20 billion in fees from these companies, which go directly to the General Partners (Phalippou et al. 2018, 581).

Second, debt is loaded onto the company through the initial deal but does not stop there. One method of securing profit is dividend recapitalizations, whereby further debt is taken on against the company, which is then used to pay dividends directly back to the investors. A PE consortium paid $21 billion for Hospital Corporation of America in 2007, of which the investors themselves paid $4.5 billion (the rest was leveraged on the company). In 2010, they recapitalized and paid themselves dividends of $4.25 billion, almost recouping their investment in one move (Appelbaum and Batt 2020, 22). In 2021, over $30 billion was paid out in dividend recapitalizations in Europe, and $82.2 billion in the USA (S&P Global 2022).

Third, valuable assets will be sold. A common method is ‘sale and leaseback’, selling off the company property (stores, warehouses) to have the company rent it back. The windfall from the sale goes to investor dividends, while the company is burdened with new rental costs, impacting profits, and frequently contributing to later bankruptcies (Appelbaum and Batt 2018). The same can occur with intellectual property (IP), whereby the valuable IP is turned into a separate legal entity, out of reach of creditors, sometimes to the point where the original company holds little more than debt (Batt and Morgan 2020, 95–6).

LBOs are common in many sectors. Retail is popular because retail companies often own valuable property that can be sold for dividends. PE investments in US healthcare grew from under $5 billion in 2000 to $100 billion in 2018 (ibid. 93), the consequences of which are detailed below. One summary case study demonstrates the nature of extractive LBOs (ibid. 91–2):

Mervyns’ Department Store was an iconic regional chain in California with a strong reputation for good value and positive community relations through its foundation. PE firm, Sun Capital, bought the chain from Target in 2004 in a leveraged buyout for $1.2 billion (with one-third equity, leaving $800 million in debt). Sun immediately sold off the real estate of the chain, paid itself back its $400 million in equity, and required Mervyns to lease property at inflated rates. It then loaded the company with more debt to pay itself and its investors a dividend. It closed some lower performing stores, required a 15% across the board headcount, ended the foundation, cut staff in warehouses, and refused to honour a credit arrangement that made it possible for vendors to get advance payment to supply seasonal merchandise. The stores soon looked shabby, lacked cleanliness, and the chain declared bankruptcy when the 2008 financial crisis hit. In fact, Mervyns’ revenues were in the black that year — at $64 million — but it owed $80 million in rent on the property it used to own. A total of 30,000 workers lost their jobs, while PE investors walked away with millions in four years.

## The Human Rights Impacts of the PE Model

This section characterizes key human rights impacts under international human rights instruments, the substantial aspect of the corporate responsibility to respect human rights. We find that LBOs pose risks to material rights such as housing and healthcare, workers, and to the right to social security through harm to pension funds.

### Rights-Relevant Goods and Services

Human rights–related goods and services include healthcare, nursing homes, housing, and private detention facilities. One tactic that has proliferated with PE involvement in US healthcare is the use of ‘out-of-network charges’, also called ‘surprise billing’, where a doctor who is not covered by the patient’s insurance (in-network) treats a patient. Generally, the patient is not informed or asked prior to incurring this cost. In 2016, 42.8% of ER visits incurred out-of-network charges. From 2010 to 2016, the average costs per patient in out-of-network charges rose from $804 to $2040. The same study found that in 2016, 86% of ER visits and nearly 82% of hospital admissions incurred surprise ambulance service bills (Appelbaum and Batt 2020). PE firms in these cases actively maximize costs to service users, failing to respect the responsibility to provide affordable essential care (CESCR, 2016a, para. 17).

Elkind and Burke (2020) describe case studies of hospital LBOs including Prospect Medical Holdings, owned by PE firm Leonard Green & Partners. Two executives each made in the region of $100 million, while hospital standards collapsed through cost-cutting measures, consistently ranking in the bottom quintile for quality of care, and on 14 occasions since 2010 were deemed by inspectors to pose ‘immediate jeopardy’ to patients. Problems cited in one LA hospital — serving a low-income community — include persistent elevator breakdowns, and a lack of medical supplies and fuel for ambulances due to unpaid bills. In a New Jersey hospital, there was a lack of personal protective equipment (PPE) during COVID-19. A doctor died who became ‘sick after being forced to reuse a single mask for four days’, and six patient deaths resulted from ‘poor infection control’ that spread COVID-19 around wards (ibid.). Such outcomes appear in prima facie breach of responsibilities toward availability and quality of care (CESCR, 2016a, paras. 12–14; 21).

PE firms have made major investments in nursing homes in the UK and the USA, attracted by the large amounts of property-owned, and dependent service users that are often ultimately supported by the state. One study found that ‘PE ownership increases the short-term mortality of Medicare patients by 10%, implying 20,150 lives lost due to PE ownership over our twelve-year sample period’. This is accompanied by declines in other measures of patient well-being, such as lower mobility, while taxpayer spending per patient episode increases by 11% (Gupta et al. 2020). In the UK, two of the largest nursing home chains (Southern Cross and Four Seasons) collapsed following PE buyouts, the former leading to 3000 job losses, while PE-owned homes paid workers on average 30% less than state-owned homes as of 2010 (Horton 2020, 151).

The same tactics occur in private detention facilities under LBOs. HIG Capita owns numerous firms that provide healthcare in correctional facilities and immigration detention. A government report described ‘untimely and inadequate detainee medical care’ at an Immigration and Customs Enforcement (ICE) Processing Center in California, where there had been 80 detainee medical care grievances filed from November 2017 to April 2018. Elsewhere, an investigation by the Department of Justice, officials learned that one HIG company had persistently understaffed the prison more than 90% of the time (PESP 2019). Nursing homes have been targeted in both the USA and UK. Property has been sold, burdening them with rental payments, and operating costs cut, resulting in lower staffing levels (Pradhan et al 2015), and care quality (Gupta et al 2020; Burns et al. 2016).

These cases implicate perhaps the clearest human rights impacts, with strong evidence of fatalities and inadequate care caused by cost cutting. Private healthcare providers hold ‘public service obligations’ to ensure affordable, universal, high-quality care (CESCR, General Comment 24, para. 18; 21). At a minimum, this includes preserving life, basic hygiene, and adequate staffing levels (ibid.). An important aspect is that rights holders are dependent upon the services. Doctors, patients, residents, and inmates cannot reasonably avoid the harmful situation, and therefore, the company holds responsibility for the human rights of these individuals.

### The Right to Housing

Perhaps the most well-known PE investment in housing is that of Stuyvesant Town in New York City (Birchall 2019a, 461; Birchall 2021a). Stuyvesant Town is the largest residential block in Manhattan comprising over 11,000 apartments. It was first purchased by PE firms Tishman Speyer and BlackRock in 2006. The total price of $5.4billion was comprised of $1.9 billion raised from fund investors, $112.5 million each from Tishman Speyer and BlackRock, and roughly $3.3 billion in loans leveraged against the apartments. Stuyvesant Town was previously almost exclusively rent-controlled. The rule is that rent control lasts only as long as the existing tenant stays. There are other ways in which rent can be increased, such as through renovations to the property, all with various restrictions.

The deal only made economic sense if rent could be increased rapidly. They planned to increase the rate at which rent-controlled tenants left and to increase the rents in deregulated apartments. They did this through liberal interpretations of eviction law, ‘shower[ing] hundreds of tenants with eviction notices’ and of the rule that rental prices can be increased more rapidly after renovations (Bagli 2015). They failed. In 2010, they could not pay the $16.1million monthly mortgage fee and defaulted. At the time of the default, they also owed residents over $200 million following legal claims over evictions and rent increases (Appelbaum et al. 2013, 510). These compensation claims were never met due to the default, despite Tishman Speyer alone holding ‘$2 billion in cash at the time of the default’ (Appelbaum et al. 2013, 511). This is made possible through the strategic division of assets through the separate legal entities principle under company law. By utilizing law to reduce their functional ability to provide compensatory remedy, PE firms risk breaching their responsibility to provide remedy for harm caused (UNGPs, Principle 22).

The volume of PE investment is a major issue in and of itself. Hard numbers are difficult to come by, but PE has been buying up global housing at a tremendous rate. PE firms were responsible for 85% of Freddie Mac’s 20 biggest deals financing apartment complex purchases by a single borrower. Although, as PE firms like to note, each firm does not own major proportions of housing stock, ‘[a]cross the top 20 markets where Blackstone owns single-family rental homes, Blackstone’s acquisitions on average represent less than 1% of all housing sales’ (Blackstone 2021), the volume is growing significantly every year, with investors buying one in seven housing units in the USA in 2021 (Schaul and O’Connell 2021). This trend started with the global financial crisis and continues to boom as more investments are sought.

Such investments in housing have mechanistic effects on access to housing. It is a zero-sum game where profit must be generated from higher housing costs. The major profit centres in rental housing are those pockets of large, affluent cities in which lower residents still reside (Farha 2017). Two UN human rights mandate-holders described that ‘in neighbourhoods heavily invested by PE firms… more than 7,400 families and individuals are evicted every day’ (Deva and Farha 2019, 3). Where tenants have a degree of protection, various tactics are used such as strict payment schedules with harsh penalties for failure, and poor quality maintenance designed to push low-income residents out (ibid.). It is important to note that while all landlords may have an incentive to charge as much as possible, only large investors, in practice mainly PE firms, have the legal knowledge and the management structure to fully exploit every opportunity, pushing the regulations to their outer limit, and, as in the Tishman deal, beyond.

Businesses near certainly breach their responsibility toward the right to housing through such tactics. Affordability is a core criterion of the right to housing (CESCR, General Comment 4, para. 8). Reducing affordability such that the local community cannot afford housing constitutes an adverse human rights impact upon this community, as may the more specific tactics such as penalty regimes (ibid., para. 17).

A more extreme version of this strategy is known as ‘collateral stripping’, where companies deliberately defund a company to avoid liabilities (Mengden 2021). Prominent examples include J. Crew, Neiman Marcus, Cirque du Soleil, IHeartMedia, PetSmart, and Acosta. Typically, the valuable components are moved to a new corporate entity and the original company is allowed to go bankrupt, without the funds to pay creditors. J. Crew was owned by two PE firms, TPG Capital and Leonard Green & Partners, with all its assets tied up as collateral on a $1.5 billion loan. J. Crew transferred intellectual property to an unrestricted subsidiary to free it up as fresh collateral to take on further credit. In 2020 J. Crew filed for bankruptcy under the weight of a $1.7 billion debt stemming from the initial PE takeover in 2011.

### Working Conditions

LBOs cause three major threats to workers. First, LBOs may reduce access to work through downsizing and bankruptcy; second, LBOs may reduce working conditions through cost-cutting, contractual changes, and the breaking of implicit contracts with workers; third, the increased risk of bankruptcy combined with tactics to reduce liabilities means that ex-workers may be unable to access full compensation.

The right to work is potentially jeopardized by the extreme levels of downsizing and bankruptcies. A WEF study (2008) found that LBOs resulted in a 10% reduction in employment within 5 years. Severe jobs losses followed Kraft’s LBO of Cadburys’ in the UK (Batt et al. 2013, 512–3). Bankruptcies have the most severe effects. Of the 14 largest US retail bankruptcies in the last decade, 10 were owned by PE. These bankruptcies resulted in the loss of 1.3 million jobs — 600,000 jobs directly and over 700,000 jobs indirectly in connected businesses (Baker et al. 2019). In the USA, seven PE-owned grocery chains employing 125,000 workers have filed for bankruptcy since 2015 (Appelbaum and Batt 2018). Lone Star Funds LBO of Southeastern Grocers led to bankruptcy and thousands of job losses and included a loan of $475 million that was ‘used to pay dividends of $458 million to Lone Star’ and required the payment of $205 million in interest between 2014 and 2018 (ibid.).

The right to work in international law entails that states should not interfere with or take measures that reduce access to work, either for individuals (e.g. discriminatory rules) or quantitatively (e.g. adopt economic policies that reduce the availability of work). This includes that relevant caveat that states may undertake actions that reduce access to work where necessary, for example, during an economic crisis. States must prove that such measures were ‘fully justified by reference to the totality of [Covenant] rights’ (CESCR, General Comment 3, para. 9).

Applied to the corporate responsibility to respect, this suggests that businesses, within their own functional ambit, should avoid measures that reduce access to work unless absolutely necessary, and should be able to justify any such reductions, most likely on the basis of business necessity. To be clear, businesses hold no universal responsibility to provide employment for all, but they do hold a responsibility to respect the right to work of their employees, including by not endangering their jobs unnecessarily. While job cuts can be permissible, they must be justified. Therefore, not all business tactics in which profit is increased by reducing staff numbers are permitted. Where an act such as a dividend recapitalization has imperilled company health causing job losses or bankruptcy, it is unlikely to be justifiable as a necessity.

Two issues invoke the right to just and favourable conditions of work: first, declining labour standards, and second, the lack of compensation for employees after bankruptcy. Watt (2008, 560–1) cites various studies that show slower wage after LBOs and that PE board representation led to ‘management breaking implicit agreements and transferring wealth from employees to new owners’. Davis et al. (2014) find that earnings per worker fell following LBOs and that earnings fell markedly in lower-skilled sectors such as retail. Amess (2018, 18–19) finds limited aggregated evidence for this, but highlights that skilled workers often benefitted, while less skilled workers suffered. This maps contemporary concerns around decent work and increasing economic inequality. Those workers that are more replaceable, both legally and economically, suffer a regression in working conditions both in terms of pay and security (European Commission 2022, 5). Appelbaum and Batt (2014, e.g. at 211–213) provide several case studies in PE takeovers that have led to extreme cuts to labour conditions and jobs. Scheuplein (2019, 10–11) notes the significant disempowerment of labour organizations in Germany following PE takeovers.

Companies have a responsibility to avoid causing adverse impacts by reducing access to ‘just and favourable conditions of work’. Among the standards from General Comments are the gradual elimination of precarious work (CESCR, General Comment 24, para. 19) and a living wage (CESCR, General Comment 23, para. 19). Any cuts that increase precarity or cut wages below living wage level would be a *prima facie* cause of ‘impacts’. It is unclear from the evidence to what extent cuts to employment standards reach these criteria, but at least in some US states, there is the legal possibility of such cuts. As with the right to work, cuts could be permissible if necessary, but in the case of LBO, cost-cutting the primary motivation is rapid profit rather than necessity. While the overall quantitative effect of LBOs on working conditions is debated, some extreme levels of cuts are evident, and the human rights lens provides a useful means by which to disaggregate and address these cuts without prejudice to other PE firms.

Bankruptcies particularly harm creditors, including redundant workers. An LBO of grocery chain Haggen by Comvest Partners led to bankruptcy and $100 million left owed to unsecured creditors, including ex-employees (Brickley 2017). Access to justice and adequate reparations are key to all rights (CESCR, General Comment 18, para. 48; CESCR, General Comment 23, para. 57), and form the third pillar of the UNGPs, access to remedy. Bankruptcy presents a problem because the company is no longer in a position to fulfil obligations to creditors, and the bankruptcy itself cannot be prohibited. While bankruptcy itself is clearly not an inevitable human rights abuse, it may be arguable that extractive practices that lead to increased risks of bankruptcy could be within the remit of human rights due diligence on the grounds of preventing human rights risks.

### The Right to Social Security

Ironically, given that pension funds are the single largest institutional investor in PE, LBOs put pension schemes at serious risk through strategic use of bankruptcy. This is particularly true in the US, where The Employee Retirement Income Security Act (ERISA) insures pension funds. While this provides a degree of guarantee to pension recipients, it also means that during bankruptcy firms can pass their pension obligations on the state. Strategic bankruptcy entails an owner filing for bankruptcy to shed its liabilities, then reforming the company minus pension liabilities (Appelbaum and Batt 2014, 82–83). This is what Sun Capital, the PE owners of ‘Friendly Ice-Creams’, did, saving $100 million in pension liabilities (Lewis 2015). An LBO led by Morgan Stanley PE of Tops Market loaded $724 million of debt onto the company, paid $377 million in dividends, and led to a bankruptcy that withdrew unionized defined benefit plans from over 12,000 workers and replaced with (lesser) 401(k) plans (Appelbaum and Batt 2018). Lewis (2015, 6) cites that:

Since 2001, at least 51 companies have abandoned pensions in bankruptcy at the behest of PE firms. They have dumped $1.592 billion in pension bills onto a government-backed agency. Because some pension benefits are not covered by insurance, it has left some of the 101,989 workers or retirees with lost pension benefits amounting to at least $128 million.

Pensions are part of a human right rarely connected to business, that of the right to social security (Article 9 of the ICESCR). As a state obligation this requires, primarily, that states ensure that those in need have basic welfare provision, including unemployment, disability, and pensions (CESCR, General Comment 19, para. 2). The state obligation to respect includes not interfering in access to social security (ibid. para. 44). The obligation to protect requires ‘ensuring that private actors do not compromise equal, adequate, affordable, and accessible social security’ (ibid. para. 46). Taken together, the corporate responsibility to respect should at least include that businesses do not interfere with ‘equal, adequate, affordable, and accessible social security’. Taking a firm into bankruptcy to drop pension liabilities as part of an extractive profits plan that reduces pension benefits would appear to breach these standards.

## Private Equity: Defining the Problem in Human Rights Terms

We define the problem of PE as one of incentives, methods, and scale. PE firms attract investors by promising high returns. The sole aim of the manager is then to ensure that this return is met. This is the fundamental business model, the most important managerial incentive. Sometimes this return can be met simply by initiating a dividend recapitalization and the sale-and-leaseback of property. In such situations, investor expectations are met by pure value extraction. This does weaken the company long-term and may well lead to redundancies, poorer services, or bankruptcy, which may reduce access to human rights. In other situations, investor returns will require cutbacks that harm workers and/or service users, or create an inability to provide remedy. The issue of scale matters because such occurrences are becoming more common, and because at scale this model is now being applied to different markets. The oft-cited ‘housing crises’ in London, New York, and Berlin will only be made worse as this business model buys up more of the market (Potts 2020).

A traditional human rights approach grounded in justiciable claim rights might argue that the deaths and lack of care that occurs in US healthcare constitutes an adverse human rights impact, that redundancies and higher house prices generally do not and that the business strategies that caused these outcomes are beyond the scope of human rights. We do not agree that such an approach is right for this problem. A debate could be had on whether redundancies could constitute an adverse impact, linking joblessness to poverty, inadequate welfare, child hunger, and so on, citing the UNGPs on ‘reducing access to rights’, and state obligations to avoid the ‘retrogression’ of access. We will not undertake this project here (but see: Birchall 2022a; Courtis et al. 2014). Instead, we argue that the business model and the incentive structure therein is more important than specific human rights outcomes for three reasons.

First, in a world of weakening safety nets and increasing economic inequality, a business model that exacerbates joblessness and costs of essentials is causing harm to human rights. Second, if pains are taken to eliminate the most overt human rights outcomes, harmful outcomes will be pushed elsewhere. Addressing fatalities in healthcare may encourage an increase in homelessness as the investment shifts to housing. Third, this business model is booming. Only a human rights lens on the model itself can comprehensively address the harms therein.

The fundamental issue with applying human rights standards to the PE business model is to link cause and effect. The extensive use of value extraction to fund returns is a central root cause of eventual human rights harm, as is the need to deliver rapid investor value itself. The most overt human rights impacts, such as understaffed hospitals and abuse of tenants’ rights, are directly caused by this business model. Such instances are best seen as tendencies inherent in the business model. Unlike, for example, fast fashion’s reliance on cheap labour, the PE model does not cause a specific sub-set of rights issues. Rather, the model is based on reducing costs and maximizing prices in order to deliver maximal investor gains. Because PE firms invest in all types of companies, this harm may manifest as harm to workers, hospital patients, tenants, and many others. Wherever PE firms invest, they tend to take existing practices or risks to extremes. Clark (2009) contextualizes this in terms of ‘disconnected capitalism’, that PE managers are beholden only to investors, and tend to treat companies themselves as ‘disposable’, thereby making them far more willing than a traditional ‘shopfloor’ manager to harm workers and service users. The need to extract value to fund returns is part of the model, and therefore, harm is a necessary and legitimate end.

This leads to the most challenging question. Often, PE investors first extract value such as through a dividend recapitalization and then pay for that through cuts to labour protection or service provision. They voluntarily place themselves in a position where such cuts are necessary. This means that human rights protections must address these initial acts prior to the harmful outcomes, even though these initial acts are not directly human rights-related. If a choice such as a dividend recapitalization eventually causes risks to human rights, can we apply the UNGPs to this root cause?

### Applying the UNGPs to Private Equity

The UNGPs provide useful tools, particularly the ‘cause, contribute, linked to’ framework and the risk management tools (Van Ho 2021). A company contributes to an adverse human rights impact when its actions are part of a wider matrix of causes. This comes in two forms, cumulative and assistive. Cumulative impacts occur where the firm contributes a quantitative amount to a larger impact, of which emissions causative of climate change is a clear example. Assistive impacts occur when a company assists another company in causing an impact, for example, by supplying materials to a company that violates rights (Ruggie 2017; Birchall 2019b).

Dividend recapitalizations are a form of value extraction for investor profit that can weaken the company, but they do not directly cause an adverse impact. Rather, the now weakened company may have to undertake actions that harm rights-holders. Seen as such, dividend recapitalizations fit the definition of a (potential) cumulative contribution to the eventual impact. The distinction is that the contribution accumulates risk to rights within business operations, rather than as part of an accumulation across different businesses, as in the climate change example. In our reading, this is not substantive difference. A key reason that patients died in the example above was cost-cutting; a key reason the cost-cutting was necessary was value extraction. The act of value extraction therefore contributed to the eventual impact and should rightly be understood as within the scope of business responsibility. As a known contributory factor to human rights risks, they should be understood as falling under the UNGPs risk management procedures.

Human rights principles permit states to reduce access to rights where this is unavoidable, such as an economic recession, provided they do so with a view to protecting all rights as best they can (Courtis et al. 2014, 126). The UNGPs start from the ostensibly stricter premise that all adverse impacts must be prevented, mitigated, and/or remedied — that is, they are all prohibited and those impacts that do occur must be remedied to compensate for the illegitimate harm caused (Ruggie 2017). However, the UNGPs do contain various principles that suggest more nuanced responsibilities in certain contexts. The principle of ‘severity’ allows for the most serious harms to be addressed first, while ‘contributions’ to a human rights impact allow for partial responsibility based on a company’s own level of impact (Principle 24; 13).

The principle that some adverse impacts should only be mitigated, rather than fully prevented, implies a further form of nuance particularly applicable to this situation (Mares 2018, 32–34; 55–58). Mitigation applies most obviously to large scale potential adverse human rights impacts where many individuals may be affected and those effects are not perfectly determinable. Some human rights impacts, such as slavery, are never permitted. Others, such as environmental impacts, are not so absolute. For environmental impacts, some harm to the environment is permitted so long as no extreme harm occurs and the harm that does occur is necessary and minimized. Malaihollo (2021, 141–143) highlights the importance of ‘reasonableness’ and ‘good faith’ in environmental due diligence to avoid an ‘impossible or disproportionate burden’ (ibid, 141). It seems reasonable to apply a similar standard to companies causing harm through cost cutting, service reductions, or price increases. The harm to human rights should be mitigated as best as possible, but there is no absolute prohibition where the reduction in access to rights is necessary. This does not hand companies a sweeping right to ‘remove or reduce’ all forms of rights enjoyment in exchange for investor dividends. The impacts should be minimized as best as possible. This implies a strong link between cause and effect — cost-cutting that reduces access to rights to save a company during an economic downturn is permissible, while cost-cutting to fund a dividend recapitalization may not be. Taking the simple example outlined above of ambulances lacking fuel, were this an unavoidable outcome due to a genuine unavailability or economic crisis and which the hospital had attempted to mitigate as best as possible, the hospital would be blameless. In the situation described above, that is clearly not the case, and the hospital holds full responsibility for the adverse outcomes.

### Risk Management Through HRDD

LBOs take many forms and not all cause adverse impacts, but the practice does contain inherent risks. Here, the UNGP risk management tools, particularly HRDD, are useful, allowing risks to be accounted for without necessarily mandating sweeping prohibitions. HRDD is defined in Principle 17 as ‘a process to identify, prevent, mitigate and account for how [companies] address their adverse human rights impacts… The process should include assessing actual and potential human rights impacts, integrating and acting upon the findings, tracking responses, and communicating how impacts are addressed’. Businesses are required to understand both their actual and potential adverse human rights impacts (human rights risks). HRDD ‘should be initiated as early as possible in the development of a new activity or relationship’ (Principle 17). HRDD ‘includes assessing the human rights context prior to a proposed business activity… and projecting how the proposed activity and associated business relationships could have adverse human rights impacts on those identified’ (Principle 18). HRDD should be undertaken ‘prior to major decisions or changes in the operation’, including ‘market entry, product launch, policy change, or wider changes to the business’ (Principle 17). The HRDD standard has been translated into binding legislation in several European countries, particularly in France, Germany, and Norway. It also features in the draft EU directive on corporate sustainability due diligence, published by the European Commission in February 2022. All these instruments are loosely modelled after the UNGPs HRDD principles.

Human rights risks should be prevented and/or mitigated as relevant. Mitigation requires ‘actions taken to reduce [an impact’s] extent, with any residual impact then requiring remediation’ (Principles 17 and 22; OHCHR 2021, 7). Further, ‘human rights risks cannot be the subject of a simple cost–benefit analysis, whereby the costs to the enterprise of preventing or mitigating an adverse impact on human rights are weighed against the costs to the enterprise of being held to account for that harm’ (OHCHR 2021, 40). The design is simple. Ideally all human rights risks should be prevented prior to their coming to fruition. Where this is not possible they should be mitigated, and those components that cannot be mitigated should be remediated. This means that there should be no value in causing human rights impacts.

The responsibility that companies should project how a ‘proposed activity… could have adverse human rights impacts’ and that HRDD should be undertaken ‘prior to major decisions’ would appear to capture decisions such as land sell-offs and dividend recapitalizations. These are strategically planned business acts based on financial due diligence, on which PE firms pride themselves. The economic impact of a specific dividend recapitalization on the firm should be predictable, including the extent of cost-cutting or pricing increases. By identifying those cuts or price increases that will potentially cause adverse human rights impacts, those impacts can be eliminated or mitigated prior to the dividend recapitalization, for example, by reducing its quantitative value. It is not technically difficult to apply HRDD to LBOs, or subsequent actions such as dividend recapitalizations. A company performing adequate HRDD would do its utmost to ensure that its hospitals were clean and reasonably resourced, for example. To comply with the UNGPs, PE managers should undertake HRDD prior to any major ‘extractive’ act, including LBOs, dividend recapitalizations, sell-offs, and collateral stripping to understand the likely impact on rights-holders, and any cutbacks that they implement should have the adverse impacts prevented and/or mitigated.

To finish, the paper makes three recommendations to embed meaningful HRDD within PE operations. First, as an analytical issue, it is vital to reify economic causes of risks to human rights. Business decisions, such as to extract value for investor profit, reshape companies and can cause human rights risks just the same as operational risks, such as health and safety in the workplace. The human rights risks can be and should be mapped through HRDD just like any other. There should not be, as there appears to be in practice currently, a divide between operational risks and risks that stem from business decisions.

Second, to make HRDD meaningful in the PE sector and around business decisions more generally, public compliance with human rights standards is key. This entails that any potential root cause of risk, such as an LBO, has its human rights risks mapped in published documents so that stakeholders can understand their risk management processes (UNGPs, Principles 16 and 18). This brings practices that are currently backgrounded into the foreground and allows third parties to understand more about the business model and its risks. It also places the onus on the PE firm to layout the likely effects of any decision, a necessary starting point in a complex business environment. In addition, it would allow governments to respond better to the risks. If HRDD reports incorrectly analyse the risks to rights, wrongly predicting minimal harm, for example, it demonstrates that stricter mandatory rules may be needed.

Third, PE firms have a responsibility to provide remedy. Given the elevated risk of bankruptcy and the known problem of collateral stripping, PE firms should set aside capital to ensure that if a human rights risk from an economic decision is realized, it can remedy that harm. It should be a minimalistic ethical principle that the risk of such decisions be analysed, stopped if the risk is too high, and that insurance policies are put into place. If PE firms refuse to do this voluntarily, governments should consider mandating a rule or caveating the separate legal entities principle to allow for remedy. This can be done through robust due diligence legislation and accompanying regulation. Arguably, states even have an obligation to act in this respect under the state duty to protect against human rights abuse as articulated in the UNGPs.

The aim of the UNGPs is not to stifle business and these risk management procedures do not prima facie prohibit any action. They ask only ‘what is the likelihood of this action causing an adverse human rights impact?’ If an LBO or a specific dividend recapitalization or sell-off does not cause any risks, it is permissible. If a company can demonstrate that the risks have been accounted for and prevented adequately, all such actions are still permitted. For critics of PE this may be a powerful tool, allowing for a delineated focus on risks and harmful practices without prejudice to the value creative form. Indeed, it is likely that applying HRDD to PE activities will be more immediately fruitful as a critical lens on PE than as a practicable change to PE. PE firms are well aware of the risks of their tactics but remain singularly focused on investor returns. Indeed, this is the root of the problem. PE firms must be solely focused on investor value. Until the human rights risks of their business model are publicly challenged, there is minimal incentive for them to change.

## Conclusion

The UNGPs have not previously been applied to the PE business model. This is a major gap because PE firms control huge amounts of business activity and they utilize common ‘value extractive’ means to generate profit. Applying human rights principles to business models and the business decisions that underlie eventual harm to human rights is an important endeavour which contributes to ensuring comprehensive respect for human rights within business. Applying the UNGPs to PE can help to align investor decisions with the UNGPs and to critique investors from a human rights perspective. This article has argued that the UNGPs apply to PE investments, that serious adverse human rights impacts occur as a result of these investments, and that the protective principles of the UNGPs, particularly HRDD, can usefully be applied.

We strongly recommend that states drafting mandatory HRDD laws consider how they will apply to the PE business model. The three recommendations above should be the starting point. Economic actions such as LBOs that cause no direct harm but increase risks later on must be part of the HRDD analysis. The mapping of the risks of such actions must be public so as to be open to outside analysis. Remedy must be made available for harm caused by these actions. At their most insidious, PE firms appear to be asset-stripping whole sections of the economy, as in US grocery chains (Appelbaum and Batt 2018) and exploiting the most desperate human needs in health services (Appelbaum and Batt 2020). Only a tiny group of investors reap the benefits of this harm. Regulating such business practices is a human rights necessity and increasingly even a developmental necessity for states as poverty and deprivation rise.

There is need for further research into the precise application of HRDD to such business practices as this has only been sketched at a conceptual level here. Particularly, how a PE firm is meant to evaluate the human rights risks of, for example, a specific dividend recapitalization, could be debated. The paper has also not addressed the importance of the ‘socially-binding’ aspect of the UNGPs (Ruggie 2020) and the need for normative pressure in these less obvious areas of human rights risk. PE firms are not overly responsive to public pressure and the complexities through which they operate make it difficult to generate tangible demands. Nonetheless, PE is a vast industry with vast influence, including over rights-holders, and that provably can generate severe adverse impacts as a part of its business model. It is hoped that this initial foray into possible human rights responsibilities may generate further interest in applying the UNGPs to business models with a focus on root causes beyond overt violations.
